# Knowledge, Attitude, and Practice of Teledentistry in Periodontal Diagnosis Among Dental Interns at a College in Sebha, Libya: A Cross-Sectional Questionnaire Study

**DOI:** 10.7759/cureus.58330

**Published:** 2024-04-15

**Authors:** Adel M Mohamed, Fatma Ahmed, Khaled M Gondi, Khaled A Salem, Omar B Mohammed, Syed Wali Peeran

**Affiliations:** 1 Prosthodontics, Faculty of Dentistry, Sebha University, Sebha, LBY; 2 Faculty of Dentistry, Sebha University, Sebha, LBY; 3 Preventive Dental Sciences, Jazan University, Jazan, SAU

**Keywords:** telemedicine (tm), teleperiodontics, teledentistry, teleconsultation, sabha, sebha, periodontal diagnosis, libya

## Abstract

Background

Teledentistry, a subspecialty of telemedicine dedicated to dentistry, has shown promise in improving access to dental care, particularly in rural and isolated areas. It integrates digital and telecommunication technology with dentistry, allowing for the remote distance exchange of relevant clinical information and digital dental imaging for dental consultation and treatment planning. Periodontal disease diagnosis is crucial for effective treatment and prevention of irreversible loss of periodontal structures. Early identification of periodontal disease can be pivotal in preventing periodontal tissue destruction and tooth loss and improving the overall quality of patients' lives. Sebha is a city located in the Fezzan region of southwestern Libya. It is the capital of the Sabha District and the Sabha Governorate. The city is situated in the Libyan part of the Sahara desert and is known for its strategic location as a gateway to the Sahara desert. However, there is a lack of information on the use of teledentistry in Libya in general and the use of teleperiodontics, especially in periodontal diagnosis. Hence, the aim of this questionnaire study was to evaluate knowledge, attitudes, and practice of teledentistry among dental interns at Sebha, Libya.

Materials and methods

A paper-based questionnaire consisting of 28 close‑ended Likert scale questions, including sections assessing the knowledge, attitude, and practice of teledentistry and teleperiodontics, was administered to dental interns at the Faculty of Dentistry, Sebha University, Sebha, Libya.

Results

The study surveyed 42 dental interns of the Faculty of Sebha, Libya, in total, with an 82.35% response rate among them. The majority of participants (59.5%) felt that teledentistry is reliable in arriving at periodontal diagnosis. The majority of participants (64.3%%) also had acceptable levels of trust in teledentistry equipment. However, over 45% percent of dental practitioners voiced their worries about patient privacy. Most of the participants suggested using teledentistry in some form in their future practice.

Conclusion

Teledentistry and its branch teleperiodontics are recent developments and its penetration among dental healthcare workers, and their knowledge, attitude, and practice remain to be thoroughly understood. The changing trends in attitudes and practice as a consequence of changes in Internet and technological awareness and the effects of the pandemic warrant closer observation and study.

## Introduction

Teledentistry is a branch of telemedicine. It has gained significant attention in the field of dentistry, for remote dental screening, diagnosis, consultation, and treatment planning [1]. It combines digital and telecommunication technologies with dentistry to facilitate the remote exchange of relevant digital imaging and clinical data for treatment planning and consultation [2]. Teledentistry is of extensive use in various disciplines within dental practice, including for periodontal disease diagnosis [3]. Further, it has been recognised as a valuable tool for improving access to dental care and oral health outcomes [4].

Periodontitis is a prevalent inflammatory disease affecting the tooth-supporting tissues, induced by dysbiotic bacterial biofilm on tooth surfaces [5]. The risk factors for periodontitis are diverse and include lifestyle factors such as smoking and alcohol consumption [6]. Studies have also shown that periodontitis can lead to an increase in blood pressure, thereby increasing the risk of arterial hypertension [7]. Moreover, a higher risk of cardiovascular illnesses is linked to chronic infections such as periodontitis [8]. Furthermore, there is a significant association between periodontal disease and head and neck cancer, with a recent meta-analysis demonstrating an odds ratio of 3.17 [9]. Further, individuals with periodontal disease have a higher risk of developing oral cancer, with the severity of periodontitis correlating with the appearance of oral squamous cell carcinoma [10]. Moreover, periodontal disease has been associated with a sevenfold increase in the risk of giving birth to a preterm, low-birthweight baby in women with periodontitis compared to those without periodontitis [11,12]. Periodontitis is a multifactorial disease and is associated with various systemic conditions. Therefore, it is essential to consider the gravity of these factors in the prevention, diagnosis, and management of periodontitis.

Sebha is a city located in the Fezzan region of southwestern Libya. It is the capital of Sabha Governorate, Libya. The city is situated in the Libyan Desert and is known for its strategic location as a gateway to the Sahara desert [13]. Sebha has a rich history dating back to prehistoric times, with an indication of human habitation in the area dating back to prehistoric times [14]. Telehealth was launched in Libya recently [15,16]. Teledentistry, in general, and teleperiodontics, in particular, can be practical tools that could be employed widely to reach the patient remotely and could enormously help in screening and diagnosing periodontal disease, as well as motivating patients for better oral hygiene, reminding patients of their dental and periodontal appointments, thereby improving periodontal health of the patients.

There is a scarcity of scientific literature regarding the use of teledentistry in general and teleperiodontics in particular among dental healthcare workers in Libya. Hence, this is the first attempt, to the best of our knowledge, to study the knowledge, attitude, and practice of teledentistry in periodontal disease among dental interns in Sebha, Southern Libya.

## Materials and methods

A cross-sectional survey study was carried out among dental interns in the Faculty of Dentistry, Sebha University, Sebha, Libya, to know the knowledge, attitude, and practice of teledentistry especially in relation to periodontal diagnosis and teleperiodontics. The paper-based questionnaire was administered to all the dental interns. Participation in the survey was voluntary, and verbal consent of the participants was taken. The questionnaire was modified from an earlier study by Penmetsa et al. [17].

The questionnaire consisted of seven sections. The first section was about the gender of the participant. The second section dealt with the knowledge about teledentistry. The third section deals with periodontal diagnosis, while the fourth and fifth are about attitudes towards teledentistry and attitude towards teleperiodontics, respectively. The sixth and seventh sections are about the practice of teledentistry and the practice of teleperiodontics, respectively. The questionnaire consisted of 28 Likert-scale closed-ended self-administered questions. The questionnaire was designed based on the available scientific literature and was validated by Penmesta et al. [17]. The protocol for the study was approved by the scientific committee and ethical board of the faculty of dentistry at Sebha University in Sebha, Libya. The data were entered in MS Excel and analysed.

## Results

The paper-based questionnaire was administered to all the dental interns. The college has 51 dental interns, out of whom three are males and 48 are females. Forty-two interns participated in the survey, among whom 39 (92.9%) were females and three (7.1%) were males (Table 1). The ages of the participants were similar as they were among the same batch of dental interns from the same college.

**Table 1:** Gender distribution of the sample

Gender	N	Percentage
Female	39	92.9
Male	3	7.1
Total	42	100.0

Table 2 shows knowledge about dentistry. Over a quarter of dental interns have heard about teledentistry (26.2% (heard and often heard)), while 57.1% (never heard and did not hear) did not (Q-1). Additionally, 50% of dental interns did not agree or disagree with the utility of teledentistry in monitoring the patient's oral health (Q-2). A vast percentage of the sample also did not feel that a specialist could be assessed via teledentistry (Q-3).

**Table 2:** Knowledge about teledentistry

Sl No.	Question	Options	N	Percentage
1	Have you ever heard about teledentistry?	Never heard	15	35.7%
		Did not hear	9	21.4%
		Not sure if heard	7	16.7%
		Heard	5	11.9%
		Often heard	6	14.3%
2	Teledentistry can be used to monitor a patient's oral health	Strongly agree	-	-
		Disagree	13	31.0
		Neither agree nor disagree	21	50.0
		Agree	7	16.7
		Strongly agree	1	2.4
3.	Can access to a specialist in Dentistry be increased using teledentistry?	Never	7	16.7
		Rarely	10	23.8
		Sometimes	12	28.6
		Often	11	26.2
		Always	2	4.8

Table 3 reveals the knowledge and attitude of dental interns towards periodontal diagnosis through digital communication. Using digital diagnosis, 28.6% of dental interns were able to differentiate between a healthy and diseased periodontium (Q4). In addition, according to 21.4% of respondents (Q5), digital communication technologies can be used to diagnose periodontal diseases. In the meantime, 59.5% of respondents believed that teledentistry could reliably identify periodontal disease (Q8).

**Table 3:** Periodontal diagnosis through digital communication

Sl No.	Question	Options	N	Percentage
4	Using digital diagnostic evidence, can you differentiate a healthy periodontium from a diseased periodontium:	Very unlikely	8	19.0
		Unlikely	14	33.3
		Neutral	8	19.0
		Likely	10	23.8
		Very likely	2	4.8
5	Periodontal conditions can be diagnosed using digital communication methods:	Strongly agree	2	4.8
		Disagree	20	47.6
		Neither agree nor disagree	11	26.2
		Agree	9	21.4
		Strongly agree	-	-
6	Will you be able to detect the presence of periodontitis using teledentistry?	Very unlikely	7	16.7
		Unlikely	19	45.2
		Neutral	7	16.7
		Likely	9	21.4
		Very likely	-	-
7	Do you feel the need to consult a Periodontist for a second opinion to diagnose and manage a Periodontal condition?	Never	6	14.3
		Rarely	4	9.5
		Sometimes	15	35.7
		Often	7	16.7
		Always	10	23.8
8.	How reliable do you think teledentistry is in making Periodontal diagnoses?	Absolutely reliable	3	7.1
		Very reliable	7	16.7
		Reliable	15	35.7
		Somewhat reliable	7	16.7
		Unreliable	10	23.8

Table 4 demonstrates the attitude of dental interns towards teledentistry. Furthermore, 64.3% felt that their trust in equipment used in teledentistry was within the range of acceptable accuracy (Q-9). A majority (52.3%) of dental interns also had a positive attitude that underserved communities can access dental care via teledentistry (Q-12).

**Table 4:** Attitude towards teledentistry

Sl No.	Questions	Options	n	Percentage
9.	Do you trust the accuracy of the equipment used in teledentistry?	Very accurate	3	7.1
		Accurate	11	26.2
		Acceptable accuracy	13	31.0
		Somewhat accurate	3	7.1
		Not accurate	12	28.6
10.	Teledentistry can be a convenient form of performing dental check-ups:	Strongly agree	3	7.1
		Agree	11	26.2
		Neither agree nor disagree	11	26.2
		Disagree	15	35.7
		Strongly disagree	2	4.8
11.	Teledentistry will be an excellent adjunct to regular dental check-ups:	Strongly agree	7	16.7
		Agree	13	31.0
		Neither agree nor disagree	7	16.7
		Disagree	14	33.3
		Strongly disagree	1	2.4
12.	Teledentistry can be used to access underserved communities for their dental needs	Strongly agree	3	7.1
		Agree	19	45.2
		Neither agree nor disagree	13	31.0
		Disagree	6	14.3
		Strongly disagree	1	2.4

Attitudes of the subjects surveyed towards teleperiodontics are shown in Table 5. The survey participants (47.6%) show a degree of confidence in diagnosing periodontal conditions using teledentistry (Q-13). Less than a quarter (21.5%) show a likelihood to support their periodontal diagnosis obtained via teledentistry (Q-14). On the one hand, only 9.6% agreed (Q-15) that teleperiodontics will be sufficient to start the treatment until the patient visits a dental clinic. On the other hand, a majority of the interns feel that teleperiodontics can be an efficacious means of delivering supportive periodontal care (Q-16) and motivating patients to improve their oral hygiene (Q-17). A majority also thought that, in the foreseeable future, teleperiodontics cannot substitute conventional periodontics as a means for reaching a periodontal diagnosis (Q-18).

**Table 5:** Attitude towards teleperiodontics

Sl No.	Questions	Options	n	Percentage
13.	Are you confident in diagnosing periodontal conditions using teledentistry?	Very confident	4	9.5
		Confident	11	26.2
		Somewhat confident	5	11.9
		Not confident	13	31.0
		Never confident	9	21.4
14	Can you support your provisional diagnosis obtained through teledentistry?	Very unlikely	1	2.4
		Unlikely	19	45.2
		Neutral	10	23.8
		Likely	10	23.8
		Very likely	2	4.8
15.	Teleperiodontics will be enough to initiate the treatment until the patient visits a dental clinic.	Strongly agree	7	16.7
		Agree	15	35.7
		Neither agree nor disagree	9	21.4
		Disagree	9	21.4
		Strongly disagree	2	4.8
16	Teleperiodontics can be an effective method in providing Supportive periodontal therapy:	Strongly agree	2	4.8
		Agree	19	45.2
		Neither agree nor disagree	10	23.8
		Disagree	10	23.8
		Strongly disagree	1	2.4
17.	Can teleperiodontics be an effective method in motivating the patient to improve his oral hygiene?	Strongly agree	14	33.3
		Agree	17	40.5
		Neither agree nor disagree	5	11.9
		Disagree	5	11.9
		Strongly disagree	1	2.4
18.	Do you think, in the near future, teleperiodontics could replace conventional periodontics in terms of periodontal diagnosis?	Very unlikely	15	35.7
		Unlikely	11	26.2
		Neutral	6	14.3
		Likely	9	21.4
		Very likely	1	2.4

Table 6 deals with the practice of teledentistry, while Table 7 deals with the practice of teleperiodontics. Over 40% of the survey participants felt that teledentistry would save time and cost for the dentist and the patient (Q-19), while over 45% felt that teledentistry could breach patient privacy (Q-20). In addition, a majority of the participants thought that they would practice teledentistry at least in some form (Q-23). It was also felt that teledentistry could be used as a teaching aid for maintaining proper oral hygiene (Q-26). A majority also aim to recommend dental hygiene products and drugs to treat periodontal diseases using teleperiodontics until an in-office visit (Q-27), while the majority agree with reevaluation via teleperiodontics of periodontal treatment (Q-28).

**Table 6:** Practice of teledentistry

Sl No.	Questions	Options	n	Percentage
19.	Teledentistry saves cost and time for the dentist and the patient	Very unlikely	5	11.9
		Unlikely	10	23.8
		Neutral	10	23.8
		Likely	13	31.0
		Very likely	4	9.5
20	Teledentistry will violate patient privacy	Strongly agree	4	9.5
		Agree	15	35.7
		Neither agree nor disagree	14	33.3
		Disagree	7	16.7
		Strongly disagree	2	4.8
21	Have you ever received any patient digital diagnostic details for consultation via social media?	Never	8	19.0
		Rarely	10	23.8
		Sometimes	10	23.8
		Often	5	11.9
		Always	9	21.4
22	Have you ever sent any patient diagnostic details for consultation using social media?	Never	10	23.8
		Rarely	5	11.9
		Sometimes	13	31.0
		Often	11	26.2
		Always	3	7.1
23.	In the future, will you practice teledentistry?	Never	7	16.7
		Rarely	12	28.6
		Sometimes	9	21.4
		Often	8	19.0
		Always	6	14.3

**Table 7:** Practice of teleperiodontics

Sl No.	Questions	Options	n	Percentage
24	Have you ever diagnosed a periodontal condition through digital communication media?	Never	15	35.7
		Rarely	11	26.2
		Sometimes	9	21.4
		Often	5	11.9
		Always	2	4.8
25	If yes, did you confirm your provisional diagnosis during the in-office visit of the patient:	Never	7	16.7
		Rarely	10	23.8
		Sometimes	9	21.4
		Often	9	21.4
		Always	1	2.4
26	Teledentistry can be used as a tool for good oral hygiene maintenance training	Strongly agree	6	14.3
		Agree	18	42.9
		Neither agree nor disagree	12	28.6
		Disagree	2	4.8
		Strongly disagree	4	9.5
27	Will you prescribe oral hygiene aids and medications to manage periodontal conditions using teleperiodontics until an in-office visit?	Never	9	21.4
		Rarely	6	14.3
		Sometimes	19	45.2
		Often	4	9.5
		Always	4	9.5
28	Reevaluation after periodontal treatment can be done using digital communication methods after periodontal treatment	Strongly agree	6	14.3
		Agree	21	50.0
		Neither agree nor disagree	7	16.7
		Disagree	5	11.9
		Strongly disagree	3	7.1

## Discussion

Teledentistry is a relatively new field of dentistry and has risen concomitantly to the employment of newer digital means in medicine as telemedicine. In addition, a growing pool of scientific evidence underscores the evolving landscape of teledentistry and the changing attitudes and practices among dental professionals [2]. The use of emerging digital technologies and communication tools, along with the infusion of artificial intelligence, has transformed the way diagnosis, treatment, and follow-up are carried over in dentistry, as well as its various fields, including periodontics.

Several studies have been conducted to assess the knowledge, attitudes, and practices of teledentistry among dental professionals and students. For instance, in studies by Pradhan et al. and Penmetsa et al., they found that the majority of the participants had heard about teledentistry [17,18]. Meanwhile, in our study, the majority (Q-1) had not heard about teledentistry, which was similar to a study by Aboalshamat [19]. Libya has begun the implementation of telehealth quite recently [16,20]. Teledentistry in Libya faces a number of infrastructure and technological difficulties, such as patchy internet access, a shortage of hardware, and inadequate technical assistance and training, especially in rural locations where several mobile service providers have not established a connection [16,20]. This may be the reason why many of the dental interns in our study had not heard about teledentistry.

Another study in Saudi Arabia found that dental students and teaching staff demonstrated moderate knowledge and relatively poor practice of teledentistry despite a high degree of awareness and positive attitude [21]. This appears similar to our study, where a majority (Q-1) lacked information about teledentistry. A study in Malaysia indicated the readiness of dental professionals to engage in teledentistry practice, which appears to be similar to our study, where a majority of participants would like to practice teledentistry in some form (Q-23, Q-27) [22]. These results (Q-23, Q-27) were also similar to a study done by Aboalshamat [19]. Furthermore, a study conducted in Pakistan showed that, although the majority of dental professionals’ awareness and perception of teledentistry were unsatisfactory before COVID-19, it was currently satisfactory [23]. This is also shown in other studies where the impact of COVID-19 on dental healthcare professionals has evolved their perceptions and practices of teledentistry, suggesting a potential shift in the adaptation of teledentistry even beyond the pandemic phase [21,24,25]. This appears similar to our present study, where a majority showed a willingness to use teledentistry in their future day-to-day dental practice.

Moreover, in the study by Penmetsa et al. [17], the majority of dentists thought that the equipment used in teledentistry could be reliable, which was similar to the results of our study (Q-9) [17]. Similar to the results of our research, a survey by Mathivanan et al. [26] showed that less than half of dentists thought teledentistry was convenient (Q-10) [26]. This view may be due to the fact that monetizing consultations and diagnosis over teledentistry appears difficult across many parts of developing nations. In another study by Al-Khalifa et al., 60-70% of respondents said they were unsure about diagnostic accuracy, privacy, and technical dependability, whereas the participants in our present study thought that teledentistry was reliable (Q-8) and had acceptable accuracy (Q-9) [27]. However, the majority of our study felt that teledentistry (Q-20) violates privacy, which was similar to Al-Khalifa et al.'s(2020) study in the Kingdom of Saudi Arabia [27].

Furthermore, in a study by Pensmetsa et al., only 48.29% of dentists thought that they needed to consult a periodontist, while the rest of the study participants were able to distinguish between a healthy and a diseased periodontium using digital diagnostic evidence [17]. However, this is not similar to our study, where 28.6% were able to identify diseased periodontium (Q-4), and only 40.5% felt the need to consult a periodontist. These findings suggest that there may be a skill gap in the application of knowledge of periodontics in teleperiodontics, which needs to be further investigated.

The present study, along with the earlier scientific literature, indicates varying levels of knowledge, attitudes, and practices of teledentistry and teleperiodontics among dental professionals and students in different regions. While some demonstrate a high degree of awareness and positive attitudes towards teledentistry, there are variations in the actual implementation and practice. Teledentistry has been recognized as a valuable tool for improving access to dental care, especially during the COVID-19 pandemic, and has shown potential in various dental applications. Moreover, in vast and thinly populated regions of southern Libya, the availability and access to dental care are limited. Hence, the use of teledentistry, including teleperiodontics, in Libya is undoubtedly worth an alternative and must be investigated.

Our present study is a cross-sectional survey study that has limitations, including the inability to establish causality, measurement bias, and recall bias. Hence, the data procured should be cautiously interpreted. Moreover, this study was conducted among the dental interns in a dental faculty in Libya, and, hence, the results cannot be extrapolated to the entire country. However, the faculty of dentistry is the only dental college in southern Libya. Therefore, the results are of significance in the Libyan context, especially when describing the landscape of south Libyan teledentistry. In addition, the results also highlight a need to update the dental curriculum and education of undergraduate dentists to help them harness the benefits of digital communication and implement teledentistry and teleperiodontics. Additionally, these results offer a succinct synopsis of the knowledge, attitude, and practice ideas expressed by dental interns about teledentistry. These results also highlight the necessity for Libyan dental regulation bodies to invest more in providing appropriate information and training on teledentistry and teleperiodontics. Further studies in Libya should include dental practitioners across the country.

## Conclusions

Teledentistry and its branch teleperiodontics are recent developments and its penetration among dental healthcare workers, and their knowledge, attitude, and practice remain to be thoroughly understood. This study, to the best of our knowledge, is the first study intended to assess the attitude and practice of teledentistry, especially in periodontal disease among dental interns in Sebha, southern Libya. The changing trends in attitudes and practice as a consequence of changes in internet and technological awareness and the effects of the pandemic warrant closer observation and study.
